# Advanced laryngeal squamous cell carcinoma in an adolescent: A case and treatment considerations

**DOI:** 10.1002/ccr3.9442

**Published:** 2024-09-26

**Authors:** Courtney J. Hunter, Mauricio A. Moreno, Graham M. Strub

**Affiliations:** ^1^ Department of Otolaryngology‐Head and Neck Surgery University of Arkansas for Medical Sciences Little Rock Arkansas USA; ^2^ Division of Pediatric Otolaryngology Arkansas Children's Hospital Little Rock Arkansas USA

**Keywords:** laryngeal neoplasms (D007822), shared decision making (D000080536), squamous cell carcinoma (D002294), squamous cell carcinoma of head and neck (D000077195)

## Abstract

Pediatric laryngeal SCCa is a rare malignancy in childhood. High index of suspicion is critical to obtain timely tissue sample and diagnosis. Shared decision making is important when choosing treatment modalities for curative management, especially when working with adolescent patients.

## Graphical abstract

Here we present a case report of a 16‐year old patient who presented with what appeared to be a vocal cord hematoma after shouting at a sporting event, which after careful monitoring and eventual intervention was determined to be advanced squamous cell carcinoma. Only 16 reports of pediatric SCCa have ever been reported. This case report highlights the paucity of guidelines in children and focuses on shared decision making with both the patient and family.

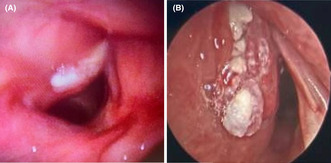

## 1 INTRODUCTION

Persistent hoarseness in a child can be caused by a wide variety of benign vocal cord lesions including nodules, hematomas, fibromas, lymphovascular malformations, and respiratory papillomas.^1^ Pediatric laryngeal malignancies are very rare, the most common being embryonal rhabdomyosarcoma, mucoepidermoid carcinoma, and synovial sarcoma.^1^, ^2^ Squamous cell carcinoma (SCCa) is a very rare disease in the pediatric larynx with only 16 cases reported in a 42‐year database review.^1^ The development of laryngeal cancer in the pediatric population has been attributed to prior irradiation, immunosuppression, HPV exposure, and/or genetic predisposition with a slight male predominance.^1^, ^3^ Guidelines are limited in management of pediatric laryngeal SCCa due to low incidence.

## 2 CASE HISTORY

The patient presented in this report is a 16‐year‐old previously healthy male who was referred to the Pediatric Otolaryngology clinic for persistent hoarseness after yelling at a sporting event 4 months prior. At the time of presentation, he was an active smoker of one pack of cigarettes per day with occasional marijuana use for approximately 1 year. In‐office flexible laryngoscopy exam at initial presentation revealed mild fullness of the left anterior‐to‐mid true vocal fold and keratin debris (clinical photo not available) Given the level of suspicion of subepithelial hematoma with the history of recent vocal trauma, speech pathology was consulted for voice trauma counseling and anti‐reflux management was initiated with daily famotidine.

**FIGURE 1:**
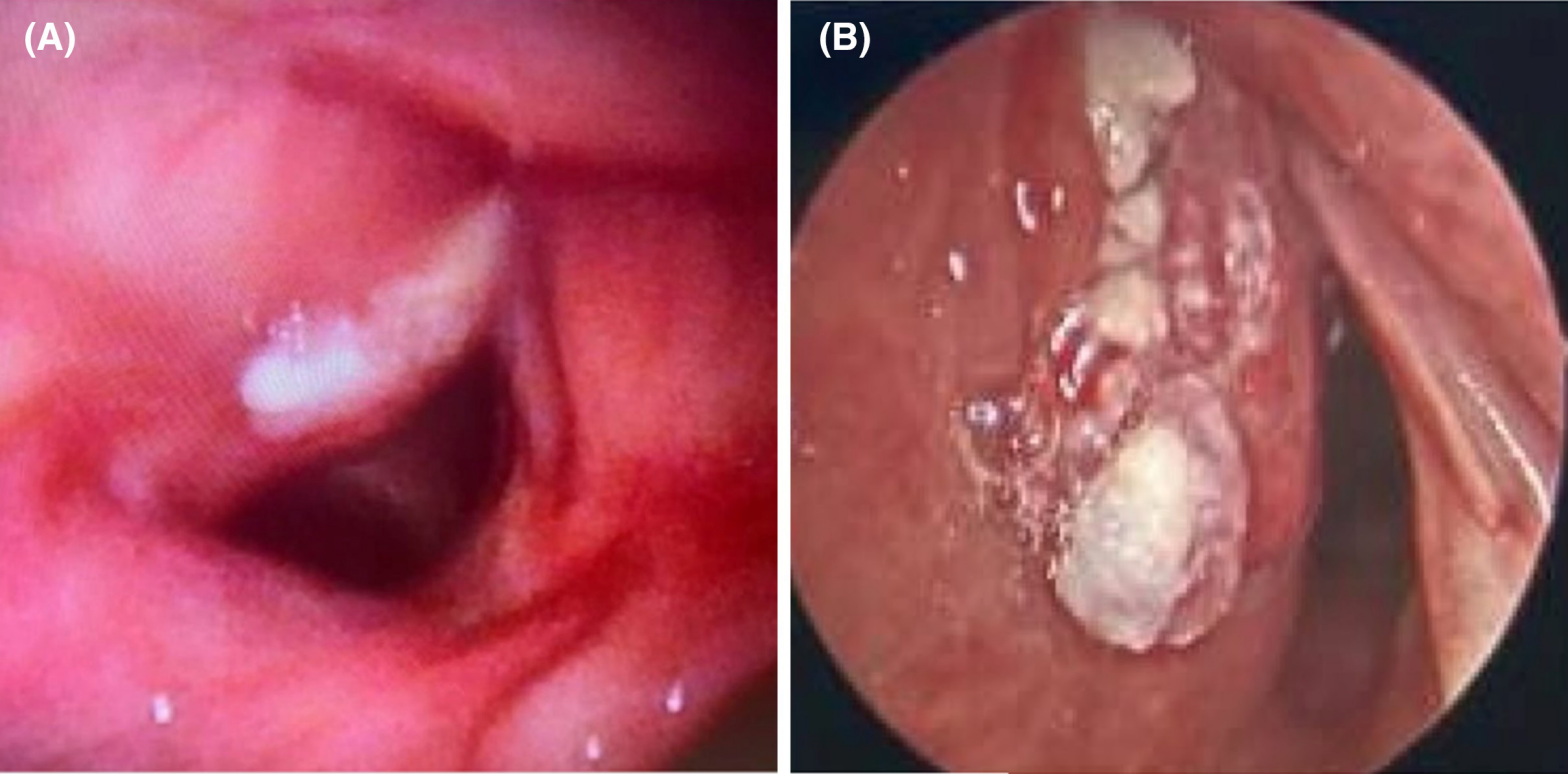
Laryngeal lesion at time of clinical exam (A) and intraoperative photo at time of biopsy (B).

The patient presented for scheduled follow up 2 months later and reported worsening hoarseness. Repeat flexible laryngoscopy revealed progressive fullness of the left paraglottic space with new leukoplakia involving the complete true vocal fold (Figure 1A) and hypomobile left arytenoid motion. Given the rapid progression of the lesion and new vocal cord motion impairment, the decision was made to proceed with imaging and biopsy.

## 3 METHODS

**FIGURE 2:**
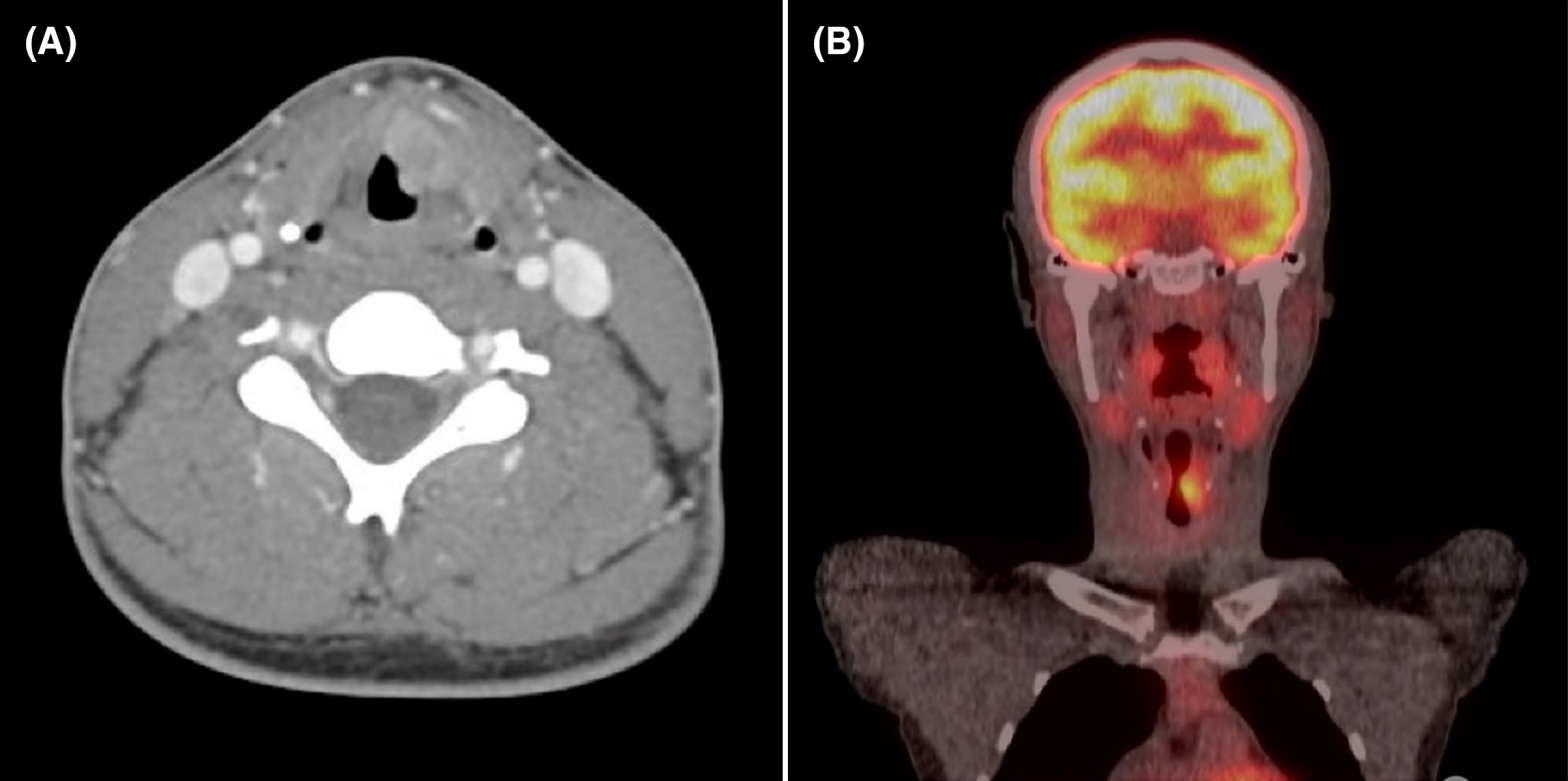
CT with contrast imaging (A) revealed enhancing lesion of the left paraglottic space extending to the anterior commissure corresponding to PET‐FDG avid lesion of the left larynx (B).

Computed tomography (CT) imaging revealed 16 × 16 × 9 mm enhancing lesion extending into the paraglottic fat without violation of the thyroid cartilage (Figure 2A). Direct laryngoscopy and bronchoscopy revealed progression of the lesion extending into the laryngeal ventricle and subglottic space (Figure 1B). The pathology revealed squamous epithelium with low‐grade dysplasia, koilocytosis, and exuberant reactive changes.

Given the highly suspicious nature of both the imaging and CT findings, the patient was referred to the Head and Neck Oncology service where repeat flexible laryngoscopy demonstrated a fixed, paramedian left vocal fold with ulceration extending into laryngeal ventricle. Repeat operative biopsy demonstrated moderately differentiated invasive SCCa. Clinical staging was consistent with T3N0M0 SCCa of the larynx, per NCCN staging system. Positron emission tomography (PET; Figure 2B) and magnetic resonance imaging (MRI) were performed as final staging imaging and did not demonstrate distant disease or laryngeal cartilage involvement, respectively.

## 4 CONCLUSIONS AND RESULTS

The patient and family met with a multidisciplinary team including a head and neck oncologist, a radiation oncologist, speech and language patholgist, and social support staff. Management options were presented to the patient and family: total laryngectomy, radiation therapy only, or chemotherapy with radiation therapy. Benefits and risks of all options were discussed, including risk of secondary malignancy with chemotherapy and toxicity of radiation therapy. The family decided to proceed with single‐modality proton radiation therapy with close follow up. The patient was treated with 6996 cGy of radiation with a total of 33 fractions. He tolerated this well with expected cervical skin changes. He did experience mild dysphagia and globus sensation during his last week of treatments. He was started on silvadine cream and a proton pump inhibitor for symptomatic management. At the conclusion of his treatment, he had significant improvement in his voice quality with only mild hoarseness.

**FIGURE 3:**
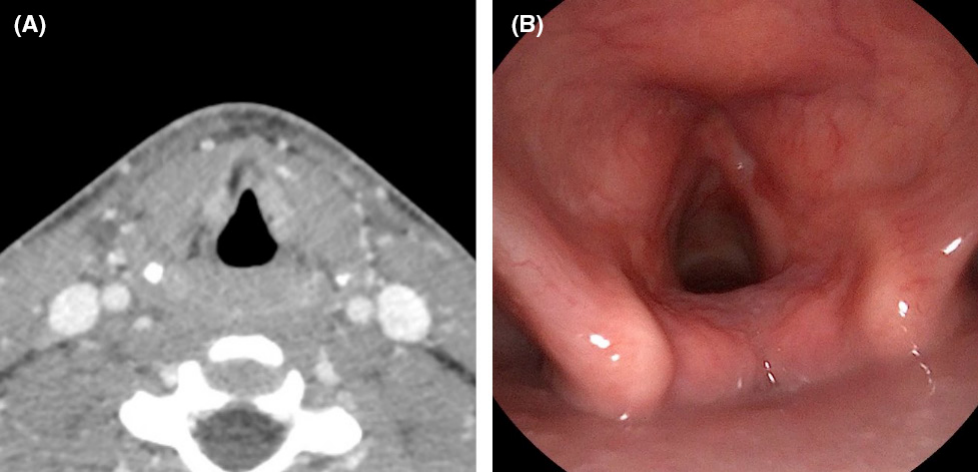
Radiographic (A) and clinical (B) evidence of complete response after treatment of laryngeal SCCa with proton radiation therapy.

He and his family presented to the head and neck oncology clinic for follow up imaging and endoscopy exam 2 months following completion of radiation therapy. During the months following his treatment, he continued to have mild hoarseness to his voice with limited pitch control. His swallowing and skin changes had resolved and he was engaging in regular social activities with his friends. His post‐treatment studies revealed clinical and radiologic remission of disease (Figure 3). He will be followed closely by the head and neck oncology team and speech pathologists. Of note, the patient had quit smoking both tobacco and marijuana products during his treatment.

## 5 DISCUSSION

Pediatric laryngeal SCCa is a very rare disease of childhood and adolescence with a mean age of diagnosis of 15 years.^1^ Given the low index of suspicion, delay in diagnosis and late stage of diagnosis has been reported in many case reports.^2^ Alternate and more insidious pathologies should be considered when the traditional timeline for benign pathologies has been surpassed. For example, in this case, the patient presented 4 months after an episode of known vocal trauma so laryngeal subepithelial hematoma was suspected though there was no evidence of late sequelae of this, including scaring or nodules on initial scope exam.^3^ Further investigation of these pathologies could have been delineated with the additional of videostroboscopy, though not always available in the comprehensive pediatric otolaryngology clinic.^3^ Limited data exists outside of the adult NCCN guidelines when managing laryngeal SCCa. Management options include local excision via laser cordectomy or hemi‐laryngectomy for early lesions to total laryngectomy for advanced lesions. Upfront or adjuvant chemotherapy and radiation therapy represent non‐surgical treatment options as well.

Toxic effects of chemotherapy and radiation therapy must be considered given pediatric risks for secondary malignancy.^1^, ^4^ Pediatric patients should be monitored for hearing loss, thyroid malignancy, and hematologic malignancy as sequelae of treatment exposure. In this case, the decision was made to proceed with primary proton therapy without concomitant chemotherapy due to risk of second primary malignancy. Proton therapy for treatment of head and neck malignancy has shown promising outcomes by sparing healthy tissue and decreasing rates of toxic side effects.^5^

A database analysis by Forsyth et al. demonstrated pediatric laryngeal SCCa has a similar or better prognosis to that of adults with a 2‐year overall survival and disease‐specific survival of 78.6%.^1^ This is in comparison to the reported rates of adult 5‐year overall survival of 51.4% and disease specific survival of 61.2%.^1^ Furthermore, Mur et al. published a systematic review which found that there was no significant difference in overall survival between surgical and non‐surgical management of advanced‐stage pediatric laryngeal masses, which contrasts with the adult data indicating a survival benefit of surgery.^2^

Careful consideration should be given to the patient's age at the time of diagnosis. Mack et al. demonstrated the importance of shared decision making between adolescent patient, family and oncologist when determining treatment plan for adolescent malignancy.^6^ A relationship with open communication was important for avoiding regret and negative psychological outcomes related to overall oncologic outcomes.^6^ The patient presented here was included in all conversations with clear expectations regarding risks and benefits of each treatment option.

## AUTHOR CONTRIBUTIONS

**Courtney J. Hunter:** Investigation; writing – original draft. **Mauricio A. Moreno:** Data curation; resources; writing – review and editing. **Graham M. Strub:** Conceptualization; data curation; formal analysis; project administration; writing – review and editing.

## FUNDING INFORMATION

None.

## CONFLICT OF INTEREST STATEMENT

All authors of this manuscript declare no conflicts of interest to disclose.

## CONSENT

Informed consent was obtained from patient's mother at the time of development of this manuscript. Written consent has been obtained.

## Data Availability

The data that support the findings of this study are available from the corresponding author upon reasonable request.
